# Protective effect of cigarette smoke on the course of dextran sulfate sodium-induced colitis is accompanied by lymphocyte subpopulation changes in the blood and colon

**DOI:** 10.1007/s00384-017-2882-9

**Published:** 2017-08-16

**Authors:** Jaroslaw Daniluk, Urszula Daniluk, Joanna Reszec, Malgorzata Rusak, Milena Dabrowska, Andrzej Dabrowski

**Affiliations:** 1 Department of Gastroenterology and Internal Medicine, Medical University of Bialystok, ul. M. Sklodowskiej-Curie 24a, 15-276 Bialystok, Poland; 2 Department of Pediatrics, Gastroenterology and Allergology, Medical University of Bialystok, ul. J. Waszyngtona 17, 15-274 Bialystok, Poland; 3 Department of Medical Pathomorphology, Medical University of Bialystok, ul. J. Waszyngtona 13, 15-269 Bialystok, Poland; 4 Department of Haematological Diagnostics, Medical University of Bialystok, ul. J. Waszyngtona 15A, 15-269 Bialystok, Poland

**Keywords:** Cigarette smoking, Immune response, Inflammatory bowel disease, Ulcerative colitis

## Abstract

**Background:**

Cigarette smoke (CS) exerts protective effect against ulcerative colitis. The mechanism of this phenomenon remains unknown. One of the possible explanation by which CS exerts its anti-inflammatory action is modulation of immune system. Therefore, the aim of the study was to evaluate the effect of CS on the course of inflammation and subpopulations of lymphocytes in the blood and colon in mice with dextran sulfate sodium (DSS)-induced colitis.

**Methods:**

C57BL6/cmdb mice were exposed to CS for 4 weeks. Colitis was induced with 3.5% DSS given for 10 days. Severity of colitis was determined by disease activity index (DAI), body weight changes, and macro- and microscopic characteristics of inflammation. Peripheral subpopulations of lymphocytes were assessed by flow cytometry (blood) or immunohistochemistry (colonic tissue).

**Results:**

Mice treated with 3.5% DSS developed severe colitis with significantly decreased body weight, increased DAI, and macroscopic and histological features of colonic inflammation. These findings were diminished after concomitant exposure to CS. Mice exposed to DSS alone demonstrated significantly decreased percentage of total CD4^+^ cells (73.1 vs. 52%, *p* = 0.0007), accompanied by increase of CD8^+^ cells (18.4 vs. 39.5%, *p* = 0.0001). Concomitant CS exposure reversed inappropriate CD4^+^/CD8^+^ ratio both in the blood and colon and significantly increased B cell presence in the colon.

**Conclusions:**

Our study has demonstrated that CS exposure decreases severity of DSS-induced colitis. This phenomenon was accompanied by changes in CD4/CD8 ratio and B cell level in the peripheral blood and colon. These mechanisms may be responsible for protective effect of smoking in ulcerative colitis.

**Electronic supplementary material:**

The online version of this article (doi:10.1007/s00384-017-2882-9) contains supplementary material, which is available to authorized users.

## Introduction

Inflammatory bowel disease (IBD) is a chronic disorder of gastrointestinal tract caused by genetic and environmental factors that affect both innate and adaptive immunological response. Two main clinical types of IBD are ulcerative colitis (UC) and Crohn’s disease (CD). Because of uncertain IBD etiology, the main goal of the current treatment is to reduce the symptoms only but not to cure the disease. Epidemiological studies showed significant increase of IBD prevalence worldwide in the last few decades [1, 2]. The most probable reason for this phenomenon is change in environmental factors like diet, smoking, drugs (i.e., antibiotics), and lifestyle alterations. Since many years, cigarette smoking has been considered as the best recognized risk factor for IBD [3]. Interestingly, smoking has a dual, completely opposite effect on development, course, and severity of two main types of IBD. Epidemiological studies have shown that current smoking almost doubles the risk of CD onset (OR = 1.76; 95%CI 1.40–2.22), while the risk for UC is significantly decreased (OR = 0.58; 95%CI 0.45–0.75) [4–6]. Moreover, smoking improves the course of UC, reduces the rate of flare-up episodes, and positively affects treatment results, and quitting smoking is considered as an independent risk factor for UC development (OR = 1.79; 95%CI 1.37–2.34) [4, 7].

Despite strong epidemiological data, underlying cellular and molecular protective mechanisms of smoking on UC course remain unclear. Currently available experimental data showed conflicting results. While some of the studies presented beneficial effect of smoking on colitis in animals, other showed aggravation of the disease [8–10]. The causes for these ambivalent results remain unknown. Cigarette smoke is a complex mixture composed of mainstream and sidestream smoke that contains more than 4500 chemicals, which are likely to interact with each other.

Recent studies have suggested that cigarette smoke affects the course of IBD by modulating the immune system. It has been shown that smoke components may change the number and function of immune cells, production of cytokines, or integrity of intestinal wall barrier [8, 11, 12]. Some reports showed significant changes in CD4 and CD8 colonic T cell function in animal model of DSS-induced colitis [13, 14]. However, the effect of cigarette smoke (CS) on these cells in mice with colitis has not been studied yet.

Therefore, the aim of our study was to evaluate the effect of CS on the course of intestinal inflammation and differences in complete blood count (CBC) and subpopulations of lymphocytes in the blood (CD4+, CD8+, CD19+) and colon (CD4+, CD8+, CD20+) in animal model of DSS-induced colitis.

## Materials and methods

### Animals

All animal experiments, including cigarette smoke exposition and colitis induction, were performed in the Center of Experimental Medicine (CEM) animal care facility, Medical University of Bialystok, according to EU Directive 2010/63/EU and approved by the Local Ethic Committee for Experiments with the Use of Laboratory Animals, Bialystok, Poland. Eight-week-old male C57BL6/cmdb mice, a strain developed in CEM, were kept in air-conditioned, pathogen-free room with stable temperature (22 °C) and with 12-h dark/light cycle. Animals were housed in sterilized cages (five animals per cage) with free access to water and standard rodent chow diet through the whole experimental period.

### Cigarette smoke exposure

After 10 days of adaptation period, mice were exposed to cigarette smoke (20 cigarettes per session, number of animals, *n* = 20) or sham treatment (ambient air, number of animals, *n* = 20) for 90 min, once a day, 5 days a week, for 4 weeks with the use of microprocessor-controlled cigarette smoking apparatus (TE-10, Teague Enterprises, Woodland, CA, USA) (Suppl. Fig. 1). To generate smoke, we used commercially available filtered Marlboro Red Box cigarettes (10 mg of tar and 0.8 mg of nicotine). We preferred Marlboro to Reference (3R4F) cigarettes since the latter are not used by the general public, and they do not contain the 600 potential harmful additives included in commercially available cigarettes. Cigarettes were smoked using the standard Federal Trade Commission method (a 2-s, 35-cm^3^ puff, once a minute for a total of nine puffs per cigarette). To simulate “active smoking,” sidestream (85%) and mainstream (15%) smoke was mixed and metered into the rodent exposure chamber. A group of sham-treated animals was exposed to room air.

### Induction of dextran sulfate sodium colitis

Eighteen days after beginning of the experiment, half of the animals both from the CS (*n* = 10) and sham treatment group (*n* = 10) were treated with 3.5% of dextran sulfate sodium (DSS, molecular weight 40,000, TdB, Uppsala, Sweden) dissolved in drinking water for 10 days to induce colon inflammation resembling human UC (Suppl. Fig. 1). The dose and duration of DSS treatment were based on literature data [15]. Consumption of water and water with DSS was determined on daily basis for each cage. Body weight of each mouse was measured on the first and the last day of experiment. Animals were monitored daily, and the activity of DSS-induced colitis was determined with the use of previously described disease activity index (DAI), which included the following variables: weight loss, stool consistency, and rectal bleeding [16, 17]. The range for each variable was between 0 and 4, with the maximal total score of 12 points.

### Sample collection

The day before the termination of the experiment, animals were exposed to CS or sham treatment on the regular schedule, and the next day without any further smoke exposure, mice were sacrificed by cardiac puncture under general anesthesia. Obtained blood samples were divided into two parts for CBC and flow cytometry analysis. Large intestine was completely removed and washed in sterile saline, and the measurement of its weight and length was performed. Both small and large intestines were stored for further histological evaluation.

### CBC and flow cytometry analysis of isolated cells

To determine CBC, 200 μl of the peripheral blood was taken. Peripheral subpopulations of lymphocytes were assessed using flow cytometry. Blood samples were treated with RBC lysis buffer (Sigma-Aldrich) for 10 min at room temperature, and the remaining cells were washed twice with cold PBS and centrifuged at 1200 rpm for 10 min. Cells were stained with the appropriate combinations of the following antibodies: FITC–anti-CD3e (145-2C11; BD Pharmigen), APC–anti-CD4 (MR4-5; BD Pharmigen), and Mouse T Lymphocyte Subset Antibody Cocktail with Isotype Control (BD Pharmigen) containing PE-Cy 7-anti-CD3e (145-2C11), PE-anti-CD4 (RM4-5), APC-anti-CD8a (53–6.7), Mouse B Lymphocyte Subset Antibody Cocktail with Isotype Control (BD Pharmigen) with PE-Cy 7-anti-CD45R/B220 (145-2C11), PE-anti-CD23 (RM4-5), and APC-anti-sIgM (53–6.7). Flow cytometric data were acquired using a FACS Canto II cytometer with BD FACSDiva Software v6.1.3 (BD Biosciences) and analyzed with Worksheet software (BD).

### Histology and immunohistochemistry

The guts were fixed with 10% PBS-buffered formalin, embedded in paraffin, cut sagittally into 5-μm sections, stained with hematoxylin and eosin (H&E), and examined by light microscopy (Olympus BX45) for histological analysis. For each animal, ten fields at ×100 magnification were captured randomly from the four different parts of the large intestine to quantify degree of inflammation. The histopathological score included two variables: (1) inflammatory cell infiltrate: (a) mild (infiltration of the mucosa) = 1, (b) moderate (infiltration of the mucosa and submucosa) = 2, and (c) severe (transmural infiltration) = 3 and (2) epithelial changes: (a) negative (no epithelial changes) = 0, (b) focal erosions = 1, (c) erosion and focal ulceration = 2, and (d) erosion and extended ulceration and/or granulation tissue and/or pseudopolyps = 3. The values of both variables were summed (total score from 0 to 6) to determine histological intensity of colitis (mild = 1–2 pts.; moderate = 3–4 pts.; severe = 5–6 pts.), as previously published [18].

To determine the lymphocytic infiltration, we used antibodies against CD3, CD4, CD8 T cells, and against CD209 (B cells). Following the deparaffinization and rehydration, epitope retrieval was carried out in the EnVision Flex Target Retrieval Solution (DAKO) at low pH. Endogenous peroxidases were blocked by incubating the sections in methanol and 3% hydrogen peroxide for 40 min. Next, slides were incubated with proper anti-mouse antibodies against CD3, CD4, CD8, and CD20 in 1:100 dilution for 1 h at room temperature. Visualization reagent EnVision (DAKO) was applied for 30 min followed by DAB solution for 10 min. The slides were then counterstained with hematoxylin and examined under the light microscope. The intensity of immunostaining was evaluated in random ten fields under ×20 magnification. The results were expressed as a percentage of cells with positive staining in accordance with the previously described manner [18]: < 10% positive cells—negative (0), 10–25% positive cells—mild (1), 26–50% positive cells—moderate (2), and 51–100% positive cells—marked (3). Appropriate positive and negative control staining was performed.

### Data analysis

Data were analyzed using Statistica 10 software. Statistical significance was determined by Mann-Whitney *U* test; *p* < 0.05 was considered statistically significant.

## Results

### Cigarette smoke diminishes the severity of DSS-induced colitis

First, we have determined the effect of smoking on the severity of DSS-induced colitis in C57BL6/cmdb mice. There was no difference in 3.5% DSS consumption independently of CS exposure (DSS-alone group = 19.8 ml/cage/day vs. DSS + CS group = 19.1 ml/cage/day). As it was expected, treatment with 3.5% DSS alone caused development of severe colitis. We observed significant decrease in body weight in comparison to controls (90.3 vs. 123.9% of initial body weight, *p* = 0.0005) (Table 1). Starting from the fifth day of DSS exposure alone, we observed changes in animal behavior and presence of loose stools. Gross rectal bleeding was noted at day 7. At the end of experiment, the DAI of these animals was significantly higher than control mice (7.4 vs. 0.0, *p* < 0.001) (Table 1). CS exposure alone did not affect the animals’ body weight, behavior, or bowel habits (Table 1). However, animals pre-exposed to CS and concomitantly treated with DSS had diminished severity of colitis, determined by less intense body weight loss, and significantly decreased DAI comparing to DSS alone-treated mice (3.4 vs. 7.4, *p* = 0.004) (Table 1). Also, the weight/length colon ratio (indirect indicator of colonic edema and inflammation) tended to be lower in mice treated both with CS and DSS as compared to the DSS-alone group (5.0 vs. 5.7, *p* = 0.3) (Table 1, Suppl. Fig. 2A). Macroscopic evaluation revealed no abnormalities in the mucosa of the rectum in control and CS mice. On the contrary, mice treated with DSS alone had substantial rectal edema, mucosal erythema with the presence of multiple erosions and ulcerations. These pathological lesions were significantly diminished after concomitant exposure to CS (Suppl. Fig. 2B). We also examined if CS or DSS had any effect on small intestine, but histological evaluations did not reveal any abnormalities (Suppl. Fig. 2C). This finding confirmed that 3.5% DSS, used in our experiment, affected exclusively colon, and therefore, it may resemble human ulcerative colitis.

**Table 1:** Change in body weight, disease activity index, and colon weight/length ratio in animals after induction of colitis with or without concomitant cigarette smoke exposure

Group	% change of body weight (SD)	*p* value	Disease activity index (range)	*p* value	Colon weight/length ratio (mg/cm)	*p* value
Control	123.9 ± 8.6		0		3.8	
CS	113.8 ± 1.8	0.033^a^	0	1^a^	3.4	0.248^a^
DSS	90.3 ± 1.3	< 0.001^a^	7.4 (6–9)	< 0.001^a^	5.7	0.003^a^
CS + DSS	94.4 ± 7.8	< 0.001^a^ 0.278^b^	3.4 (0–5)	< 0.005^a^ 0.004^b^	5.0	0.037^a^ 0.301^b^

Body weight change was calculated as a percentage of mouse weight difference at the beginning and end of experiment. Disease activity index (DAI) included the following parameters: weight loss, stool consistency, and rectal bleeding (range 0–4 points for each variable, with maximum score 12 points). To determine colon weight/length ratio, the organ was washed in sterile saline, following weight and length measurement. *p* < 0.05 was considered statistically significant

^a^ *p* value was determined by comparing treatment groups to the control group

^b^ *p* value was determined by comparing treatment groups to the DSS to CSS group

**Fig. 1:**
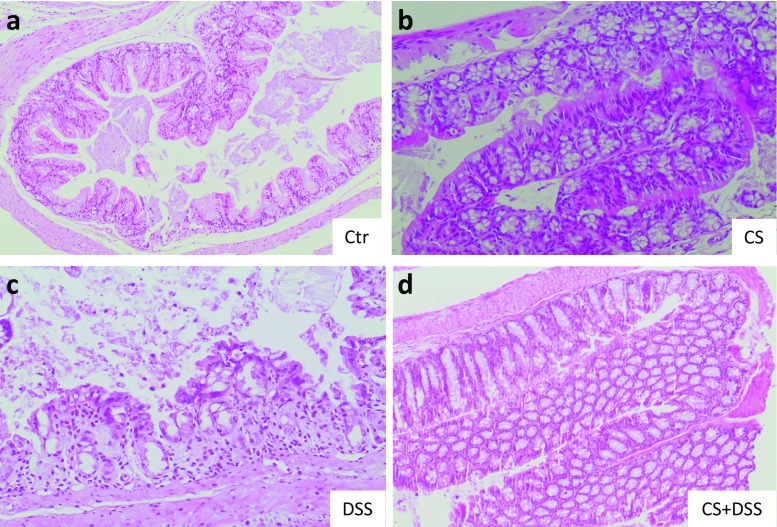
Colonic histopathological features of DSS-induced colitis. Representative H&E staining of colon sections of control mice (**a**) and mice treated with CS (**b**), DSS (**c**), and CS + DSS (**d**) (**a**, **d** 100×, **b** 200×, **c** 400×). 3.5% DSS caused severe mucosal damage and infiltration by polymorphonuclear cells and lymphocytes (**c**). Concomitant exposure to cigarette smoke ameliorated the severity of inflammation (**d**)

We did not find any abnormalities in histological picture of the colon of control or CS-exposed animals (Fig. 1a, b). However, in DSS alone-treated mice, histopathological examination of the colon showed profound signs of epithelial damage with erosions and focal or extended ulcerations (grades 2–3 in histological score), with moderate infiltration by polymorphonuclear cells and lymphocytes (localized mainly in mucosa and submucosa, Fig. 1c). Concomitant CS exposure substantially decreased the severity of histological findings in the colon with moderate mucosal and submucosal inflammatory cell infiltration (composed mostly of lymphocytes) and no or mild epithelial lesions (grade 1, Fig. 1d). Overall histological score of CS + DSS-treated mice in semiquantitative analysis was significantly lower than animals exposed to DSS alone (2.2 vs. 4.2 pts., *p* = 0.0001, Table 2).

**Table 2:** Semiquantitative analysis of histological and immunohistochemical score of mice colonic tissue after exposition to DSS or CS + DSS

	DSS	CS + DSS	*p* value
Hematoxylin and eosin			
Inflammatory cell infiltration + epithelial lesions	4.2 (4–5)	2.2 (2–3)	0.0001
Immunohistochemistry			
CD4^+^ cells	0.6 (0–1)	1.6 (1–2)	0.02
CD8^+^ cells	2.2 (2–3)	0.4 (0–1)	0.0002
CD20^+^ cells	0.4 (0–1)	1.8 (1–2)	0.002

Histopathological score included two variables: inflammatory cell infiltrate (mild—1 pt., moderate—2 pts., severe—3 pts.) and epithelial changes (negative—0 pt.; focal erosions—1 pt., erosion and focal ulceration—2 pts., erosion and extended ulceration—3 pts.). The total score range from 0 to 6. Immunohistochemical score evaluated the percentage of cells with positive staining: < 10%—0 pt., 10–25%—1 pt., 26–50%—2 pts., and 51–100%—3 pts. Sample size was ten mice per group. Data is expressed as average (min-max). *p* value was determined by comparison DSS to CSS group. *p* < 0.05 was considered statistically significant

Taking together, we have shown that active cigarette smoking had beneficial effect and ameliorated the course of DSS-induced colitis in mice both at clinical and histopathological levels.

### Effect of cigarette smoke and DSS exposure on CBC and blood lymphocyte subpopulations

There was no difference in red blood count, white blood count, neutrophils, or total lymphocyte percentage between the groups (Table 3). However, in mice with DSS-induced colitis compared to CS + DSS-treated animals, the significant reduction of hemoglobin (12.6 vs. 14.4 g/dl; *p* = 0.016), hematocrit (40.7 vs. 46.2%; *p* = 0.009), and MCV (46.1 vs. 48.5 fl.; *p* = 0.002) level was noted (Table 3). Conversely, platelets were significantly elevated in mice treated with the DSS alone as compared to the CS + DSS group (1025 × 10^3^/μl vs. 738 × 10^3^/μl; *p* = 0.001) (Table 3)**.**

**Table 3:** The effect of dextran sulfate sodium and cigarette smoke treatment on complete blood count

CBC median (min-max)	Control	CS	DSS	DSS + CS	*p*
WBC × 10^3^/μl	4.4 (2.6–6.8)	4.1 (2.0–5.47)	4.0 (2.9–17.3)	3.7 (2.9–5.0)	0.656
Lymphocytes (%)	94.9 (86.8–97.0)	93.5 (80.6–96.0)	95.0 (84.1–97.0)	93.5 (89.0–97.0)	0.456
Neutrophils (%)	4.0 (2.0–6.0)	4.6 (2.2–8.0)	4.0 (2.0–10.0)	5.0 (3.0–8.0)	0.656
RBC × 10^6^/μl	9.3 (8.8–9.9)	11.4 (10.7–11.9)	8.8 (4.5–10.1)	9.6 (8.0–11.4)	0.056
Hb (g/dl)	13.6 (12.9–14.3)	17.4 (16.8–18.2)	12.6 (6.6–14.5)	14.4 (12.5–17.9)	0.016
Ht (%)	43.6 (40.6–45.8)	55.1 (51.9–57.4)	40.7 (23.7–45.5)	46.2 (40.1–55.3)	0.009
MCV (fl)	47.0 (44.9–48.2)	48.6 (47.7–49.6)	46.1 (45.0–53.1)	48.5 (48.1–49.9)	0.002
Platelets × 10^3^/μl	841 (274–1115)	841 (586–948)	1025 (693–1277)	738 (591–839)	0.001

Sample size was ten mice per group. The data is expressed as median (min-max). *p* value was determined by comparison DSS alone to DSS + CS treatment group. *p* < 0.05 was considered statistically significant

*WBC* white blood count, *RBC* red blood count, *Hb* hemoglobin, *Ht* hematocrit, *MCV* mean corpuscular volume

**Fig. 2:**
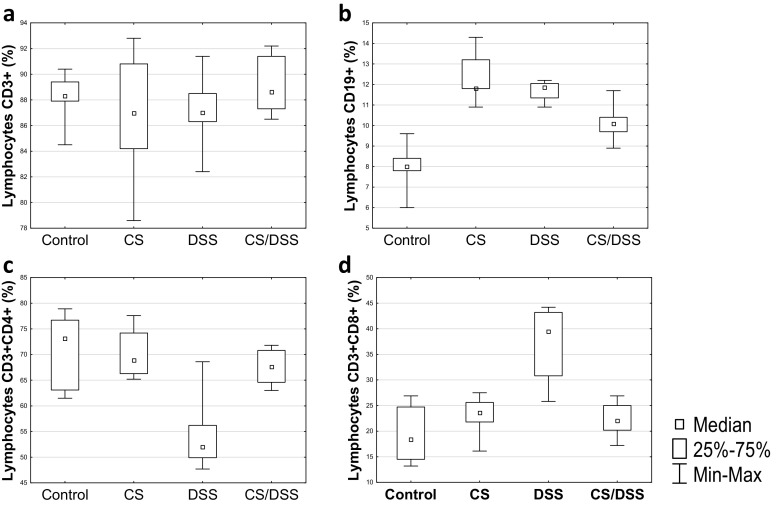
Effect of CS, DSS, and CS + DSS exposure on blood lymphocyte subsets. The percentage of T cells (**a**), B cells (**b**), total CD 4^+^ cells (**c**), and CD8^+^ cells (**d**) was determined in the blood of mice by flow cytometry (*n* = 10 mice/group). CS cigarette smoke, DSS dextran sulfate sodium

To determine the possible mechanisms involved in an anti-inflammatory action of cigarette smoke, we investigated the blood mononuclear cells by flow cytometry. No difference between study groups in percentage of blood T cells was found (Fig. 2a). However, substantial changes in B cell amount in mice were observed after colitis induction or exposition to CS. The percentage of B cells was significantly higher in mice treated with CS (median 11.8%; *p* = 0.008), DSS (median 11.9%; *p* = 0.016), or CS + DSS (median 9.8%; *p* = 0.009) in comparison to controls (median 8%) (Fig. 2b). Interestingly, despite the lack of effect of CS or DSS on total T cell number, we found substantial changes in subpopulations of T cells, namely CD4^+^ and CD8^+^. Comparing to controls, mice exposed to DSS alone demonstrated significantly decreased percentage of total CD4^+^ cells (median 73.1 vs. 52.0%, *p* = 0.0007), with concomitant increase of cytotoxic CD8^+^ cells (median 18.4 vs. 39.5%, *p* = 0.0001) (Fig. 2c, d). Conversely, combined CS exposure and DSS treatment reversed this inappropriate CD4^+^/CD8^+^ ratio, similarly to CS exposure alone. Compared to animals treated with DSS alone, amelioration of colonic inflammation by CS exposition was accompanied by significantly increased percentage of blood CD4^+^ cells (median 52.0 vs. 67.0%, *p* = 0.001) and decreased percentage of CD8^+^ cells (median 39.5 vs. 22.1%, *p* = 0.00002) (Fig. 2c, d). To summarize this part of experiment, DSS-induced colitis affected immune system with the increase of cytotoxic CD8+ cells and the decrease of CD4+ helper cells. Cigarette smoke exposure reversed this altered expression of T cell subpopulations, restoring the original balance between them.

### Effect of cigarette smoke and DSS exposure on colonic tissue infiltration by lymphocytes

**Fig. 3:**
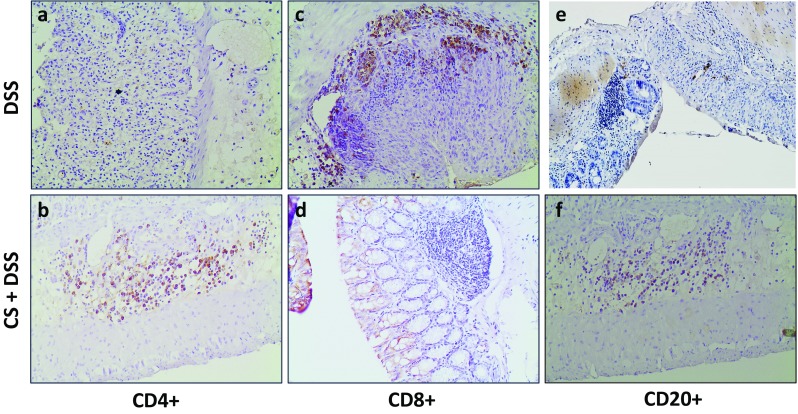
The effect of cigarette smoke (CS) and dextran sulfate sodium (DSS) on tissue infiltration by lymphocyte subsets. Immunohistochemical staining of mice colonic tissue in DSS-induced colitis with (CS + DSS) or without (DSS) concomitant cigarette smoke exposure. **a**, **b** CD4+ cells (magnification **a** ×400, **b** ×200). **c**, **d** CD8+ cells (magnification **c** ×200, **d** ×100). **e**, **f** CD20+ cells (magnification **e**, **f** ×200)

We have also evaluated if changes in lymphocytic subpopulation found in the blood were present in colonic tissue. Immunohistochemistry staining of colonic wall showed significantly decreased number of CD4^+^ cells in the DSS group in comparison to CS + DSS mice (0.6 vs. 1.6 pts., *p* = 0.02; Fig. 3a, b, Table 2). Interestingly, expression of cytotoxic CD8^+^ cells in the colon wall was significantly higher in DSS mice in comparison to the CS + DSS group (2.2 vs. 0.4 pts.; *p* = 0.0002, Fig. 3c, d, Table 2). Moreover, immunohistochemical assessment revealed less intense colonic infiltration by CD20^+^ cells in DSS in comparison to the CS + DSS group (0.4 vs. 1.8 pts., *p* = 0.002, Fig. 3e, f, Table 2).

## Discussion

Cigarette smoking is a well-known and potentially preventable risk factor for many diseases like cancer, atherosclerosis, heart disease, chronic obstructive pulmonary disease, peptic ulcer, Crohn’s disease, and many other [5]. However, it is much less appreciated that in some circumstances, smoking exerts its beneficial effect. For 50 years, we know that UC is a disease of ex-smokers and non-smokers, with the latter having threefold increased risk of developing the disease [19, 20]. Moreover, smokers are more likely to have milder course of the disease than non-smokers [6]. Current European Crohn’s and Colitis Organization (ECCO) guidelines state that “active tobacco smoking has a protective effect on the development and severity of UC” [21]. Surprisingly, in a very recent meta-analysis including 16 studies, To et al. found that the odds for colectomy, flare of disease activity, proximal extension of diseases, and the risk of development of pouchitis were not significantly lower in smokers compared with non-smokers [22]. The reason for this discrepant result remains unknown. Of course, it is highly unethical to perform human randomized trial determining the role of smoking in UC. Because of that, it is very important to develop good, reliable animal model focusing on this issue, with the use of cigarette smoke rather than its single component.

Our results have strongly suggested that smoking exerted beneficial effect on the severity of colitis (decreased DAI, weigh/length colon ratio, diminished the intensity of mucosal inflammation) in animals treated with DSS. These findings were in accordance with the Montbarbon et al. report, who also showed improvement of colonic inflammation after CS exposure [8]. However, in this study, the beneficial effect of smoking was restricted only to clinical determinants (activity score, weight/length colon ratio), without any obvious differences in tissue histological examination. In our study, we found favorable effect of CS both in clinical and histological (depleted number of tissue inflammatory cells, decreased intensity of epithelial damage) determinants. One of the possible reasons for this discrepancy may be different experimental protocols. We pre-exposed animals to cigarette smoke for longer period of time and used higher concentration of DSS (3.5 vs. 2.5%) to induce acute severe colitis. Other studies evaluating the effect of smoking on development of colitis also gave conflicting results. Galeazzi and colleagues showed that in rats exposed to CS for 2 weeks, induction of colitis by dinitrobenzenesulfonic acid (DNBS) has caused aggravation of inflammation, both macroscopically and histologically [9]. These results are opposite to our findings, most probably because the DNBS model is more related to human Crohn’s disease than to ulcerative colitis [15]. We have used DSS, because it has some advantages in comparison to other chemical substances in induction of colitis, like simplicity in use and possibility to control the degree of disease severity by changing DSS concentration or molecular weight [13, 23]. It has been shown that DSS-induced colitis is the most reliable model corresponding to the ulcerative colitis in humans [24].

One of the key unresolved questions is what constituent of tobacco smoke may be responsible for its beneficial effects on disease course. Cigarette smoke is a complex mixture of components suspended in vapor and particulate phases, and currently, we are unable to determine what chemical ingredients of tobacco smoke have “protective” effect in some diseases. It is well known that cigarette smoke apart from metabolic and genetic disturbances also affects both innate and adaptive immune response [5, 11, 25]. Verschuere et al. studied the effect of cigarette smoke on immune cell composition. Flow cytometry of Peyer’s patch tissue showed significant recruitment of dendritic cells, CD4^+^ cells (including Treg cells), and CD8^+^ cells without any impact on tissue damage [26]. In contrary, our analysis revealed that exposition to CS in animals without colitis had no effect on inflammatory cell infiltration in the colon (data not shown). One of the most important findings of our study was the effect of smoke on subpopulations of T cells in animals with DSS-induced colitis. Previous study demonstrated that severe ileitis was correlated with decreased number of regulatory CD4 T cells in the lamina propria and mesenteric lymph nodes [14]. It was also reported that smoking increases the number of CD4^+^ cells in the peripheral blood in Caucasians [27]. In our study, for the first time, we have shown that cigarette smoke reversed unfavorable CD4^+^/CD8^+^ ratio both in blood and colonic tissue during active inflammation. It is highly likely that reduction of cytotoxic CD8^+^ cell number with concomitant increase of CD4^+^ cells may be responsible for beneficial effect of smoking on severity of colitis. Moreover, our results revealed significant increase of B cells after CS exposure in animals with DSS-induced colitis. It has been shown that mice exhibited more severe colitis in the absence of B cells in the colon, and transfer of B cells attenuated the disease [28]. This phenomenon may be also implicated in protective influence of smoking in ulcerative colitis.

Our study has some limitations. First of all, we used an animal model of IBD, and our results cannot be directly transferred to the population of human with UC. People with UC are exposed to many factors that may potentiate or ameliorate the course of the disease. Moreover, they have different genetic background. Being aware of these limitations, in our experiment, we tried to reflect the real-life situation. To do that, we induced colitis with DSS which according to the previous studies can be used as a relevant model for the translation of mice data to human disease [29]. We exposed animals to the mixture of mainstream and sidestream smoke to simulate active smoking, with the use of commercially available cigarettes instead of research ones [10, 30]. In the future, we are planning to compare our results in the population of patients with UC, both smokers and non-smokers. We did not perform in vitro functional studies to evaluate the activation of lymphocyte and cytokine production after CS exposure. Moreover, in our experiment, we evaluated only acute but not chronic phase of colitis. It would be also interesting to determine if pharmacological treatment may interact with beneficial effect of CS.

In conclusion, CS exposure has attenuated DSS-induced acute inflammation in the colon. This phenomenon was accompanied by significant changes in immune cell compositions both in the blood and colon, including increase of CD4^+^ and B cells and decrease of cytotoxic CD8^+^ cells. It is possible that these effects were involved in protective action of smoking in experimental ulcerative colitis. Currently, there are many gaps in our knowledge about immune mechanisms involved in the development of inflammatory bowel diseases. We believe that findings of our study will improve understanding of the potential therapeutic targets in the immune system in IBD. However, further work is needed to fully identify the cigarette smoke substances and host-related factors (i.e., immune cells) responsible for their beneficial effect, to extrapolate these results into the clinical practice.

## Electronic supplementary material

Supplementary Fig. 1 Scheme of cigarette smoke exposure and induction of colitis in C57BL6/cmdb mice. Animals were exposed to cigarette smoke (*n* = 20) or sham treatment (*n* = 20) for 4 weeks. After 18 days of experiment, half of the animals in each group were treated with 3.5% DSS dissolved in drinking water to induce colitis. At the end of week 4, animals were sacrificed and tissue specimens were collected for further examination. (PPTX 34 kb)

Supplementary Fig. 2 Cigarette smoke decreased the severity of DSS-induced colitis in mice. a. DSS treatment resulted in a shortening of the colon length in comparison to controls, CS only or CS + DSS exposed animals. b. DSS caused severe inflammation in the rectum with the development of erythema, erosions and ulcerations, which were ameliorated by cigarette smoke. c. Histological evaluation of distal part of ileum revealed no abnormalities after DSS or CS treatment. (PPTX 29007 kb)
