# Supplementary figures and images for: Protective effect of cigarette smoke on the course of dextran sulfate sodium-induced colitis is accompanied by lymphocyte subpopulation changes in the blood and colon
Source: Int J Colorectal Dis. 2017 Aug 16;32(11):1551–9. doi: 10.1007/s00384-017-2882-9 (PMC5635083; doi:10.1007/s00384-017-2882-9)

## Slide 1
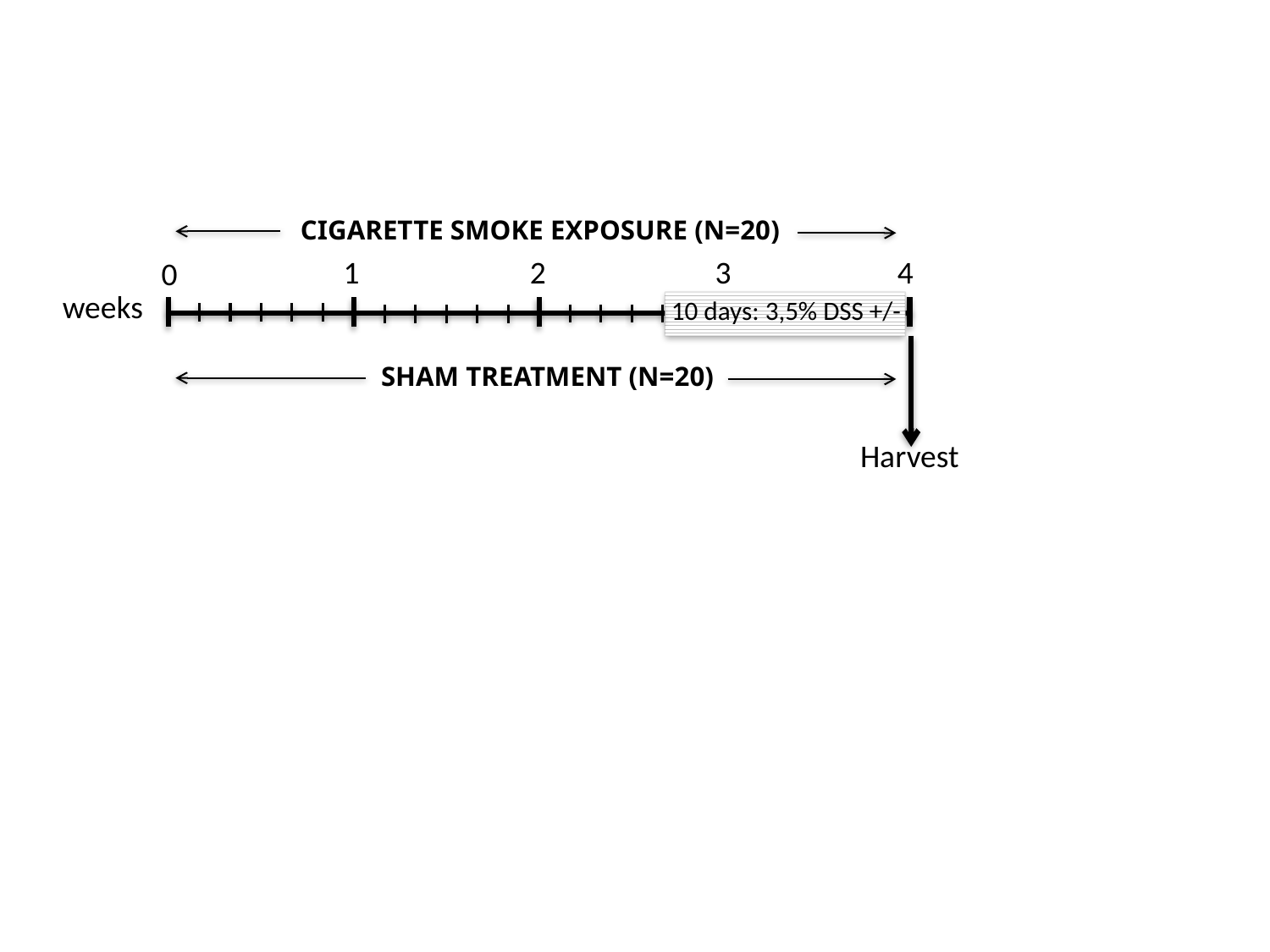

CIGARETTE SMOKE EXPOSURE (N=20)
1
2
3
4
0
weeks
10 days: 3,5% DSS +/-
SHAM TREATMENT (N=20)
Harvest

Supplement: Supplementary file 1 — Scheme of cigarette smoke exposure and induction of colitis in C57BL6/cmdb mice. Animals were exposed to cigarette smoke (n = 20) or sham treatment (n = 20) for 4 weeks. After 18 days of experiment, half of the animals in each group were treated with 3.5% DSS dissolved in drinking water to induce colitis. At the end of week 4, animals were sacrificed and tissue specimens were collected for further examination. (PPTX 34 kb) [file 384_2017_2882_MOESM1_ESM.pptx]

## Slide 1
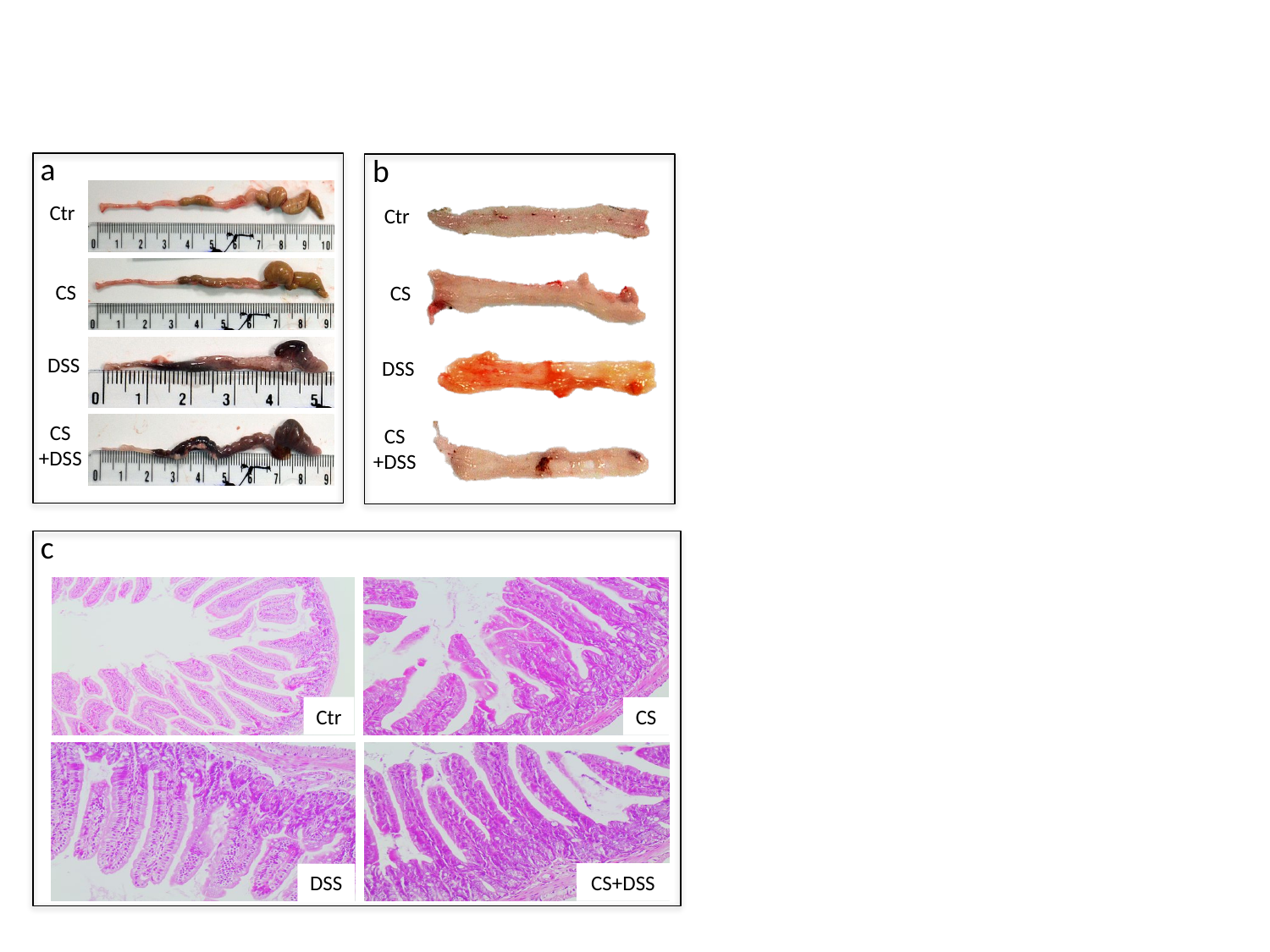

a
Ctr
CS
DSS
CS
+DSS
b
Ctr
CS
DSS
CS
+DSS
c
Ctr
CS
CS+DSS
DSS

Supplement: Supplementary file 2 — Cigarette smoke decreased the severity of DSS-induced colitis in mice. a. DSS treatment resulted in a shortening of the colon length in comparison to controls, CS only or CS + DSS exposed animals. b. DSS caused severe inflammation in the rectum with the development of erythema, erosions and ulcerations, which were ameliorated by cigarette smoke. c. Histological evaluation of distal part of ileum revealed no abnormalities after DSS or CS treatment. (PPTX 29007 kb) [file 384_2017_2882_MOESM2_ESM.pptx]
